# Suicide Trends Among Indian Institutes of Technology Joint Entrance Examination (IIT JEE) and National Eligibility cum Entrance Test (NEET) Aspirants: A Comparative Study of Demographic and Situational Factors

**DOI:** 10.7759/cureus.85812

**Published:** 2025-06-11

**Authors:** Devesh Gupta, Rajesh Ranjan, Meenakshi Singh, Chandan Kumar, Abhinav Kumar, Bhawna Kathuria, Tripti Srivastava

**Affiliations:** 1 Neuropsychopharmacology, Institute of Human Behaviour and Allied Sciences, Delhi, IND; 2 Epidemiology, National Institute of Health and Family Welfare, New Delhi, IND; 3 Obstetrics and Gynaecology, Lady Hardinge Medical College, New Delhi, IND; 4 Business Administration, Indian Institute of Management (IIM), Shillong, IND; 5 Dermatology, Madhubani Medical College, Madhubani, IND; 6 Pediatrics, Veer Chandra Singh Garhwali (VCSG) Government Institute of Medical Sciences and Research, HNB Teaching and Base Hospital, Srinagar, IND

**Keywords:** academic stress, aspirants, competitive exams, mental health, suicide

## Abstract

Background

Academic stress among students preparing for competitive exams like the Indian Institutes of Technology Joint Entrance Examination (IIT JEE) and the National Eligibility cum Entrance Test (NEET) in India has been increasingly associated with mental health challenges, including suicidal ideation. These two groups represent the largest segments of students preparing for highly competitive exams in India, with distinct academic and societal expectations, making their comparison crucial for targeted interventions. Understanding the demographic profiles, methods of suicide, and temporal trends among these students is crucial for targeted intervention and policy formulation.

Methods

This cross-sectional study analyzed data from 80 documented suicide cases among IIT JEE and NEET aspirants. Data were sourced from documented reports, such as police records and newspaper articles, to ensure reliability. Demographic information, including age, gender, and native state, along with exam preparation details and suicide characteristics, such as method used, presence of suicide notes, and number of prior attempts, were collected and analyzed. Statistical analyses included descriptive statistics, chi-squared tests, odds ratios (OR), and logistic regression to identify significant associations.

Results

The majority of suicides (81%; n=64) occurred among students aged 15-20 years, predominantly male (77.2%; n=61), and preparing for NEET (73.4%; n=58). Hanging was the most common method (75.9%; n=60), followed by poisoning (5.1%; n=4) and jumping (3.8%; n=3). Suicide notes were found in 22.8% (n=18) of cases. Students with fewer than one prior suicide attempt were significantly more likely to be from Rajasthan (OR 28.00; p<0.0001), as were cases occurring after 2022 (OR 6.05; p<0.0001).

Conclusion

This study highlights alarming rates of suicide among IIT JEE and NEET aspirants in India, emphasizing the need for targeted mental health support and intervention strategies. The findings underscore the role of academic pressure and geographic variability in suicide risk among these students. Future research should focus on understanding the underlying causes of geographic differences in suicide rates and explore region-specific interventions. Tailored mental health programs addressing local socio-cultural and educational challenges could play a crucial role in mitigating suicide risks in these vulnerable populations.

## Introduction

In India, academic excellence is deeply ingrained, especially in higher education, where exams like the Joint Entrance Examination (JEE) and the National Eligibility cum Entrance Test (NEET) are critical for careers in engineering and medicine. In 2023, these exams had over 1.5 million and 1.6 million participants, respectively, with preparation starting as early as middle school and involving years of intense study and pressure [1,2]. The competitive nature and limited seats in top institutions create a high-stakes environment that significantly impacts students' mental health. The National Crime Records Bureau (NCRB) reported 12,526 student suicides in 2021, many attributed to academic pressure, highlighting the darker side of academic ambition among JEE and NEET aspirants [3]. Similarly, aspirants for the Union Public Service Commission (UPSC) Civil Services Examination face unique challenges, with prolonged preparation periods, uncertainty in results, and high societal expectations contributing to mental health challenges. Cases of UPSC aspirants experiencing depression and anxiety have been documented, underscoring the broader impact of competitive exams on student well-being.

Educational achievement is crucial for upward mobility and family honor. The National Sample Survey Office (NSSO) reports that 75% of Indian families prioritize education for securing a better future [4]. For middle-class families, admission through JEE, NEET, or UPSC signifies not just personal success but also familial pride and financial stability. Parents invest heavily, spending ₹25,000-50,000 monthly on coaching for JEE and NEET and comparable amounts for UPSC preparation [5]. This investment comes with high expectations, placing immense pressure on students. The Centre for the Study of Developing Societies (CSDS) found that 72% of students feel intense pressure to excel, with 60% fearing social ostracism if they fail, leading to a stigmatization of failure [6].

India's coaching industry, valued at ₹24,000 crore ($3.2 billion USD) in 2022, intensifies pressure by setting unrealistic expectations and fostering cutthroat competition. Students endure grueling schedules and intense study sessions, leaving little time for rest [7]. For JEE and NEET aspirants, the pressure often stems from managing complex syllabi and competing for limited seats in premier institutions. In contrast, UPSC aspirants face the challenge of navigating an extensive and unpredictable syllabus, often coupled with repeated attempts, which prolongs their mental strain. Studies show that 65% of students preparing for competitive exams experience high stress, with 42% exhibiting depression symptoms, contributing to severe mental health issues like anxiety and suicidal thoughts [8].

The rising suicides among aspirants underline the need for systemic changes to address the mental health crisis. In Kota, a coaching hub for JEE and NEET, 28 students died by suicide in 2023, marking a high point. Similarly, cases of suicides among UPSC aspirants in urban centers highlight the broader spectrum of academic stress across competitive exams. Nationally, student suicides rose 21% from 2019 to 2021, despite initiatives like helplines and counseling, indicating current measures are inadequate [9,10]. Mental health issues are stigmatized in India, hindering students from seeking help [11,12].

This study aimed to address three critical objectives regarding the mental health challenges faced by aspirants of JEE, NEET, and UPSC in India. It seeks to identify stress determinants specific to each exam, analyze suicide distributions across states, and propose strategies to address and mitigate suicide risk factors. By focusing on preventive measures and support systems, the research aimed to alleviate the mental health burden associated with competitive exams and foster a healthier academic environment for students striving for excellence.

## Materials and methods

Study design

This study was a retrospective observational analysis focusing on suicides among aspirants preparing for the Indian Institutes of Technology (IIT) JEE and NEET exams. The primary objective was to identify patterns, contributing factors, and potential preventive measures related to suicide in this demographic. Publicly available data from January 2018 to May 2024 were utilized to capture trends and insights. The retrospective design was selected to allow the use of previously documented cases, ensuring a broad temporal and geographic scope while avoiding direct interaction with affected individuals.

Study setting

The study analyzed suicide cases reported from prominent coaching hubs across India, with particular emphasis on states such as Rajasthan, Maharashtra, Uttar Pradesh, Andhra Pradesh, and Tamil Nadu, which house significant numbers of IIT JEE and NEET aspirants. Kota in Rajasthan, a recognized epicenter for competitive exam coaching, was a major focus due to its high prevalence of reported student suicides. Data from other cities and states were included to ensure that the findings reflected regional variations in demographic, social, and institutional factors influencing student well-being.

Study population and sample size

The study population consisted of students aged 15-24 years actively preparing for the IIT JEE or NEET exams. To be included, cases had to meet specific criteria, such as confirmed suicide as the cause of death, verified engagement in exam preparation, and availability of reliable reports in the public domain. Cases involving accidental deaths, substance abuse as a primary factor, or individuals who had discontinued exam preparation prior to the event were excluded. The defined criteria ensured that the study focused exclusively on aspirants under active academic pressure. A total of 80 cases were identified and analyzed, comprising 59 NEET aspirants and 21 IIT JEE aspirants. The sample was drawn from publicly available reports and represented a subset of the total number of suicides during the study period. While the reliance on secondary data limited the ability to capture unreported cases, the dataset provided a robust basis for the descriptive and inferential analyses of suicide trends in this specific population.

Data collection procedure

Data were collected through systematic searches of online and offline sources using keywords related to student suicides and competitive exams. These searches spanned national newspapers, regional news outlets, academic publications, and social media platforms. Each identified case was reviewed in detail, with demographic, contextual, and circumstantial information extracted and verified across multiple sources to ensure reliability. Cases with conflicting or incomplete data were cross-referenced with other reports, and only those with adequate documentation were included in the final analysis. Key variables extracted included age, gender, geographic location, type of competitive exam, method of suicide, and presence of a suicide note.

Secondary data sources

The study utilized a range of publicly available secondary data sources, including prominent national and regional newspapers such as The Times of India, The Hindu, Amar Ujala, and Deccan Chronicle. In-depth analyses from investigative magazines like India Today and Outlook were also included. Peer-reviewed articles accessed through PubMed and Google Scholar offered insights into the psychological and sociological dimensions of student suicides. Additionally, online searches were conducted on platforms like Google and Bing to capture relevant cases that might not have been extensively covered by mainstream media.

Statistical analysis

Data analysis was performed using IBM SPSS Statistics for Windows, Version 26.0 (Released 2019; IBM Corp., Armonk, New York, United States). Descriptive statistics, including means, medians, and standard deviations, were calculated to provide a comprehensive summary of the dataset. Odds ratios (OR) with 95% confidence intervals (CI) were used to quantify the likelihood of suicide associated with geographic location (Rajasthan vs. other states). Logistic regression analysis was conducted to identify significant predictors of suicide, adjusting for potential confounders. A p-value of 0.05 was considered statistically significant.

Ethical considerations

All data was anonymized to protect the privacy of the individuals involved. Ethical clearance was not obtained as the study relied solely on secondary data sources. However, the study adhered to international ethical standards for research involving publicly available data, ensuring sensitivity and confidentiality in the analysis and reporting of findings.

## Results

The study of suicides among JEE and NEET aspirants revealed significant findings. Most cases (64, 81%) involved 15-20-year-olds, with males accounting for 61 (77.2%) and females 17 (21.5%). NEET aspirants constituted 58 cases (73.4%) and JEE aspirants 20 cases (25.3%). Hanging (60, 75.9%) was the most common method, followed by poisoning (four, 5.1%), jumping (three, 3.8%), drowning (two, 2.5%), and burning (one, 1.3%); 18 students (22.8%) left suicide notes. Regarding previous suicidal attempts, 17 students (21.5%) had none, 11 (13.9%) had one, nine (11.4%) had two, and two (2.5%) had three (Table 1). 

**Table 1:** Demographic and suicide characteristics among JEE and NEET aspirants JEE: Joint Entrance Examination; NEET: National Eligibility cum Entrance Test

Variables	Frequency	Percent
Age group (years)		
15-20	64	81
>20	10	12.7
Mean age (years)	18.4±1.7	
Gender		
Male	61	77.2
Female	17	21.5
Exam preparation		
NEET	58	73.4
JEE	20	25.3
Method of suicide		
Fire (burning)	1	1.3
Consuming poisonous substances	4	5.1
Drowning	2	2.5
Hanging	60	75.9
Jumping from height	3	3.8
Not mentioned	9	11.4
Suicide notes		
Yes	18	22.8
No	61	77.2
Number of previous suicidal attempts		
0	17	21.5
1	11	13.9
2	9	11.4
3	2	2.5

**Figure 1:**
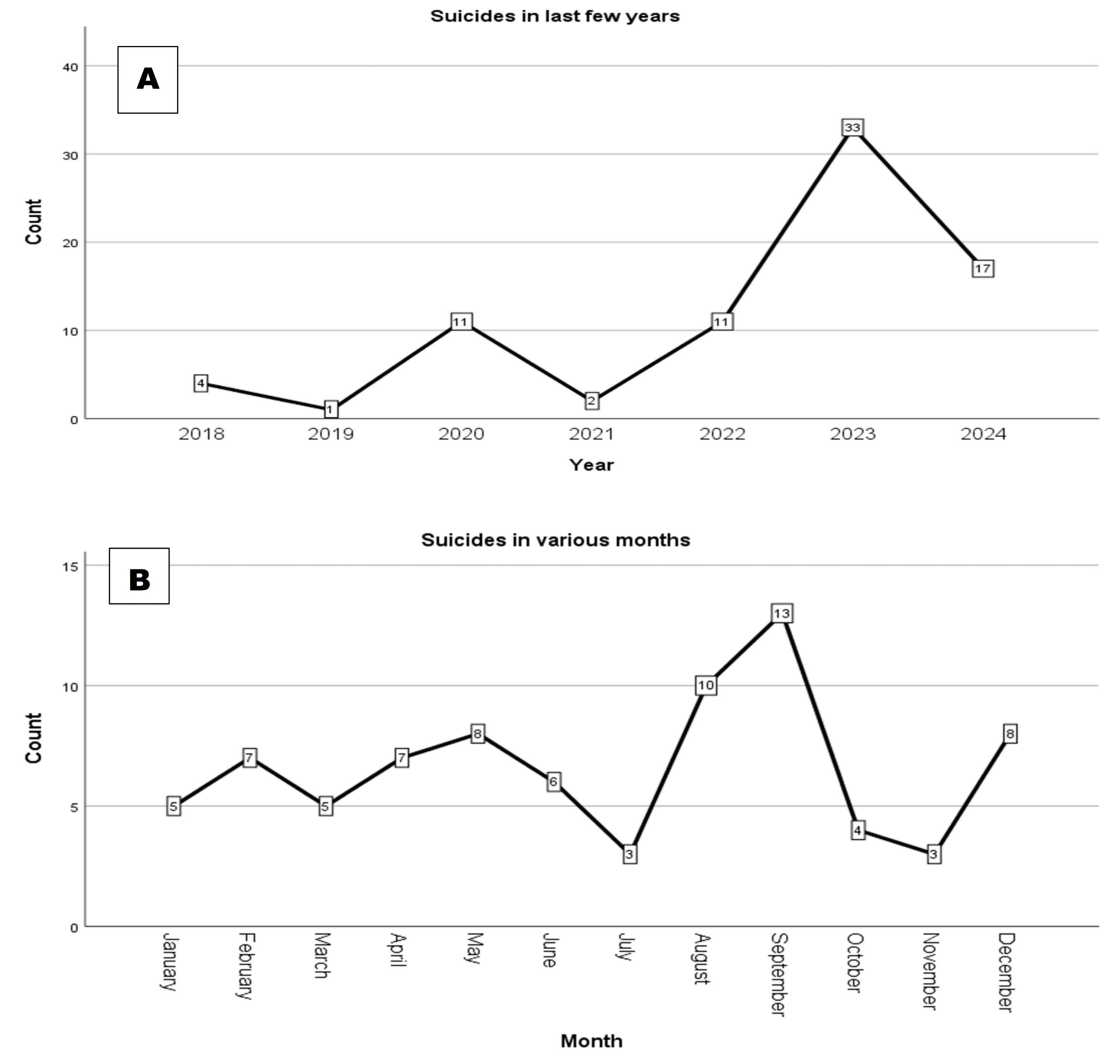
(A) Yearly distribution of student suicides among JEE and NEET aspirants. (B) Monthly distribution of student suicides among JEE and NEET aspirants JEE: Joint Entrance Examination; NEET: National Eligibility cum Entrance Test

The study found varying suicide frequencies among IIT JEE and NEET aspirants over the years, with peaks in 2023 (33 cases, 41.8%) and 2024 (17, 21.5%). Monthly, September had the highest incidence (13 cases, 16.5%), followed by August (10, 12.7%) and December and May (both eight, 10.1%). Lower frequencies were seen in 2018 (four, 5.1%), 2021 (two, 2.5%), and 2019 (one, 1.3%) (Figure 1A, 1B).

**Figure 2:**
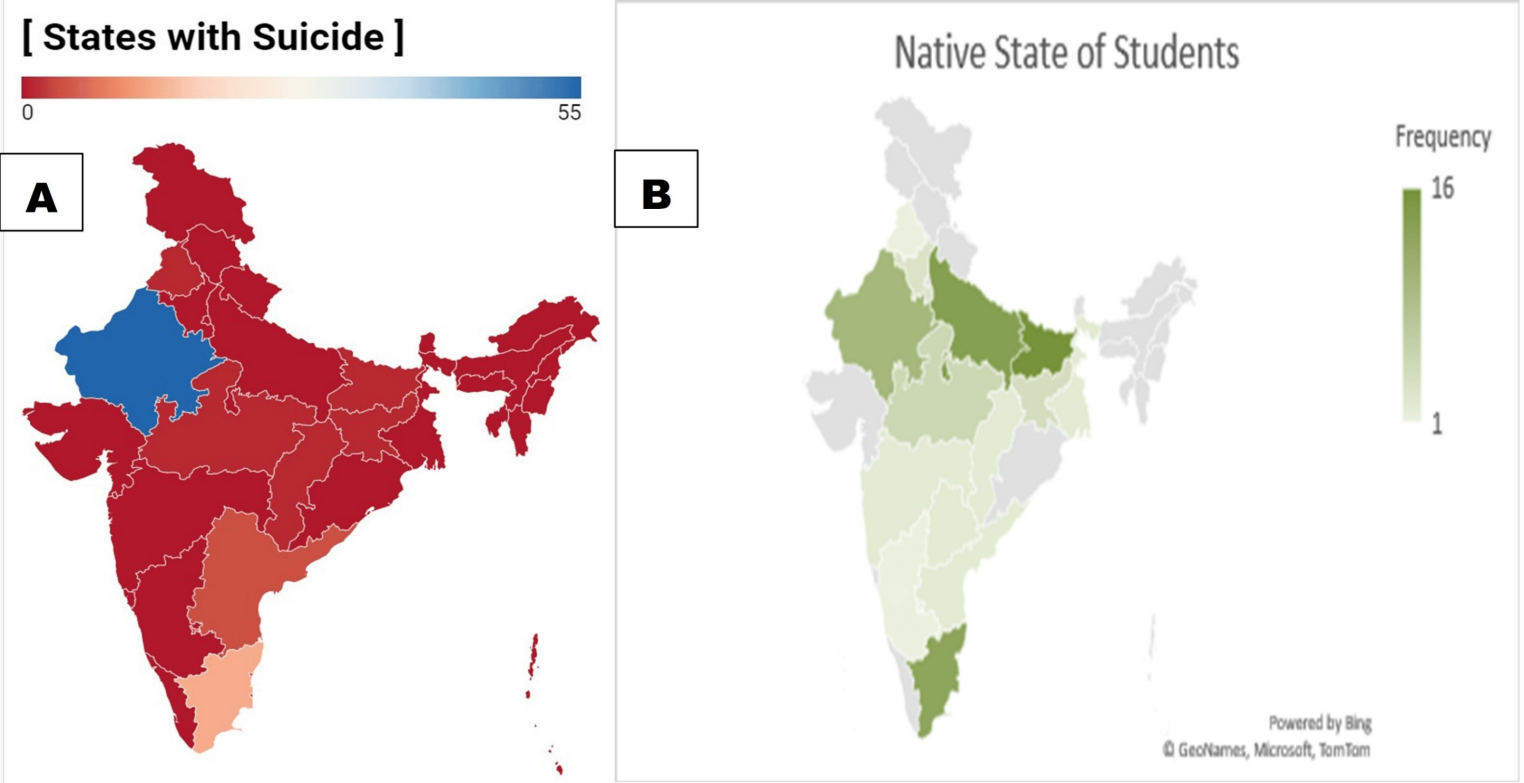
(A) Distribution of student suicides by state among JEE and NEET aspirants. (B) Native (home) state distribution of student suicides among JEE and NEET aspirants JEE: Joint Entrance Examination; NEET: National Eligibility cum Entrance Test

The majority of suicides among IIT JEE and NEET aspirants occurred in Rajasthan (55 cases, 69.6%), followed by Tamil Nadu (13, 16.5%) and Telangana (three, 3.8%). Bihar had the highest number of student suicides by native state (16, 20.3%), followed by Uttar Pradesh (14, 17.7%) and Tamil Nadu (13, 16.5%) (Figure 2A, 2B).

The analysis compared suicides among IIT JEE and NEET aspirants in Rajasthan versus other states. Age group differences were not significant (OR 1.66; p=0.464) nor were gender disparities (male OR 2.66; p=0.079). However, Rajasthan-based JEE aspirants showed significantly higher suicide rates (OR 4.82; p=0.034). Hanging was the predominant method across both regions (OR 1.56; p=0.419). Rajasthan saw significantly more suicides among first-time attempters (16, 94.1%) compared to other states (one, 5.9%) (OR 28.00; p<0.0001) and post-2022 cases (42, 84%) compared to earlier years (eight, 16%) (OR 6.05; p<0.0001), with a peak in the first half of each year (32 (84.2%) vs. six (15.8%)) (OR 3.94; p=0.01) (Table 2).

**Table 2:** Factors affecting student suicides among JEE and NEET aspirants from Rajasthan and other states JEE: Joint Entrance Examination; NEET: National Eligibility cum Entrance Test

Parameters	Rajasthan	Other states	Odds ratio (95% CI)	P-value
Age group (years)				
15-20	45 (71.4)	18 (28.6)	1.66 (0.42-6.61)	0.464
>20	6 (60)	4 (40)		
Gender				
Male	45 (75)	15 (25)	2.66 (0.87-8.15)	0.079
Female	9 (52.9)	8 (47.1)		
Exam preparation				
JEE	17 (89.5)	2 (10.5)	4.82 (1.01-22.95)	0.034
NEET	37 (63.8)	21 (36.2)		
Method of suicide				
Hanging	43 (72.9)	16 (27.1)	1.56 (0.52-4.68)	0.419
Others/not mentioned	12 (63.2)	7 (36.8)		
Suicide notes				
No	43 (71.7)	17 (28.3)	1.26 (0.40-3.91)	0.683
Yes	12 (66.7)	6 (33.3)		
Number of previous suicidal attempts				
<1	16 (94.1)	1 (5.9)	28.00 (3.10-52.47)	<0.0001
1 or more	8 (36.4)	14 (63.6)		
Year				
2023 onwards	42 (84)	8 (16)	6.05 (2.09-17.48)	<0.0001
Before 2023	13 (46.4)	15 (53.6)		
Month				
First 6 months	32 (84.2)	6 (15.8)	3.94 (1.34-11.53)	0.010
Last 6 months	23 (57.5)	17 (42.5)		

The logistic regression analysis explored factors influencing student suicides across various states compared to Rajasthan as the reference category. It revealed that the number of attempts given was significantly associated with suicide, with fewer attempts showing much higher odds, indicating heightened vulnerability among those perceiving limited chances for success. Additionally, suicides were more frequent in the first half of the year, suggesting a seasonal pattern possibly influenced by academic pressures and exam schedules. However, the type of exam preparation (JEE or NEET) and recent years (2023 onwards) did not significantly affect suicide rates compared to Rajasthan, implying that while these factors may impact other aspects of student experience, they do not independently influence suicide likelihood among aspirants (Table 3).

**Table 3:** Logistic regression analysis of factors influencing suicide among JEE and NEET aspirants from Rajasthan and other states JEE: Joint Entrance Examination; NEET: National Eligibility cum Entrance Test

Parameters	Adjusted odds ratio (95% CI)	P-value
Exam preparation (JEE)	0.10 (0.01-2.15)	0.145
Number of attempts (<1)	47.67 (2.12-106.92)	0.015
Month (first half)	24.49 (1.10-54.80)	0.043
Year (2023 onwards)	0.79 (0.05-12.48)	0.868

## Discussion

The findings of this study shed light on the complex issue of suicides among students preparing for the IIT JEE and NEET exams in India. The demographic profile of the study population revealed that the majority of suicide cases, 81% (n=234), involved individuals aged 15-20 years, with an average age of 18.4 years [13]. This demographic alignment underscores the vulnerability of young adults in their late teens, who are at a critical juncture in their academic journey, facing immense pressure to succeed in highly competitive entrance exams [14]. Adolescence and young adulthood, particularly during the late teen years, are developmental periods characterized by heightened sensitivity to stress and identity formation. The immense academic pressure faced by students in this age group, coupled with the uncertainty of their future career paths, exacerbates their vulnerability to psychological distress. The Transactional Model of Stress and Coping (Lazarus and Folkman) helps explain how these students perceive academic challenges as threats, rather than manageable stressors, leading to anxiety, depression, and, tragically, suicide.

The predominance of male suicides, 77.2% (n=223), compared to females, 21.5% (n=62), mirrors global trends in suicide rates among adolescents and young adults, where males often exhibit higher rates of completed suicides despite females typically reporting higher rates of suicide attempts [15,16]. This gender disparity warrants further investigation into the underlying socio-cultural and psychological factors influencing suicidal behaviors among male students in India [17]. Male students are often subject to societal pressures to succeed academically and financially provide for their families, leading to significant psychological strain. These pressures are further exacerbated by the intense competition associated with entrance exams. Existing mental health interventions, such as counseling programs, peer support networks, and campaigns aimed at reducing the stigma around mental health, are crucial in providing emotional support and addressing the unique challenges faced by male students.

Examining the methodological aspects, NEET aspirants accounted for the majority of cases, 73.4% (n=212), compared to JEE aspirants, 25.3% (n=73). This disparity may reflect differences in academic intensity, preparation strategies, and perceived career outcomes between medical and engineering aspirants. The pressure to excel in these exams, which determine admission into prestigious engineering and medical institutions, has led to widespread anxiety and depression among students [18]. The disparity between medical and engineering aspirants further emphasizes the need for tailored mental health interventions based on the specific pressures associated with each field.

The study also highlighted geographical disparities, with Rajasthan reporting the highest number of suicide cases, 69.6% (n=201), among the states studied, followed by Tamil Nadu, 16.5% (n=48), and Telangana, 3.8% (n=11). These findings underscore the need for region-specific interventions and support systems tailored to address varying socio-cultural contexts and educational pressures across different states in India [15]. Regional differences may reflect variations in academic competitiveness, social expectations, and regional mental health infrastructure, which necessitate focused efforts to address these disparities [19].

Furthermore, temporal trends indicated a notable increase in suicides among aspirants in recent years, with peaks observed in 2023, 41.8% (n=121), and 2024, 21.5% (n=62). This temporal clustering, particularly in August and September, may reflect broader societal changes, including increased academic competitiveness, evolving societal pressures, and shifts in educational policies affecting aspirants' mental health. August and September are critical months in the academic calendar when students face intense pressure due to final exam preparations, results, and the uncertainty of their future. This time of year can trigger emotional distress and, in some cases, suicidal ideation, highlighting the need for targeted mental health interventions during these months [20].

The finding that hanging was the most common method of suicide, 75.9% (n=219), aligns with previous studies in India, where hanging is frequently reported as the method of choice. This can be attributed to its accessibility and lethality. Additionally, cultural factors and media influences may contribute to the widespread adoption of hanging as a suicide method. In India, hanging has been portrayed in the media and public discourse as a normalized method, which may influence vulnerable individuals to perceive it as an acceptable or more accessible option. Moreover, the availability of locations suitable for hanging and the perceived ease of carrying out this method make it a common choice for students in distress [21-23].

The statistical analysis revealed significant associations between certain variables and suicide risk among aspirants. For instance, logistic regression identified that students with fewer than one previous suicide attempt were 47.67 times more likely to attempt suicide again compared to those with one or more attempts (95% CI 2.12-106.92; p=0.015). This underscores the importance of early intervention and support for students showing signs of distress or previous suicide attempts [24-27]. Identifying students at risk and providing timely psychological support can help prevent further suicide attempts and improve mental health outcomes for these vulnerable individuals.

Limitations

This study has several limitations. First, the retrospective design, relying on secondary data sources, may be prone to reporting biases and incomplete information. Additionally, the relatively small sample size of 80 may not fully capture the diversity of factors influencing suicides across the entire population of JEE and NEET aspirants. Expanding the sample size and including cases from multiple regions of India could provide a more comprehensive understanding. Furthermore, while the study provides valuable insights, it is limited by its focus on available data rather than primary sources or in-depth exploration of individual cases. Another limitation is the reliance on secondary data without direct qualitative input from the affected individuals. To gain deeper insights into the psychosocial stressors and contributing factors to suicidal ideation and attempted suicide risk among students, future research could incorporate qualitative methodologies such as interviews or focus groups with students, parents, and educators. These methods would allow for a more nuanced understanding of the personal and societal pressures faced by aspirants. Additionally, the study's cross-sectional nature does not account for changes over time. Future research should consider prospective data collection methods, tracking students over a period of time to observe how academic stress and other factors evolve and contribute to suicide risk. This would help identify critical periods or early signs of distress that could benefit from targeted interventions. Lastly, while this study provides a snapshot of suicide risk among competitive exam aspirants, it would be beneficial to explore a broader array of media reports, articles, and case studies from across the country. A more extensive examination of published materials could provide further context and detail regarding the reasons behind suicides in competitive exams, allowing for a deeper exploration of the social, cultural, and psychological factors at play.

Recommendations

To address the alarming rate of suicides among IIT/NEET aspirants in India, it is crucial to implement realistic and practical interventions that can alleviate the pressures faced by these students. Below are several recommendations.

Enhanced Counseling and Mental Health Support

Educational institutions should integrate regular mental health screenings and check-ups as part of their routine health services. This will help in the early identification of students who are at risk and ensure timely intervention. Additionally, schools and coaching centers should have dedicated counseling services staffed by trained mental health professionals. These services should be easily accessible and actively promoted to reduce the stigma associated with seeking mental health support [28].

Stress Management and Coping Mechanisms

Regular workshops on stress management, time management, and effective study techniques should be conducted to help students develop healthier coping mechanisms. Introducing mindfulness practices, yoga, and relaxation techniques as part of the curriculum can also be beneficial. These practices can help students manage stress more effectively and improve their overall well-being [29].

Curriculum and Assessment Reforms

The education system should shift from a purely exam-oriented approach to a more holistic one that values creativity, critical thinking, and overall well-being. Implementing continuous assessment methods can reduce the pressure of high-stakes exams by providing a more balanced evaluation of student capabilities. This approach can help create a more supportive and less stressful educational environment [30].

Parental and Community Involvement

Parental awareness programs should be conducted to educate parents about the pressures their children face and how to provide better emotional support and set realistic expectations. Establishing community-based support networks can also be beneficial, providing a platform where students can share their experiences and receive peer support [31].

Reducing the Coaching Culture

Regulating coaching centers is essential to ensure they do not add undue pressure on students. Encouraging self-study by promoting its benefits and providing resources for independent learning can reduce the dependency on intensive coaching. This approach can help create a more balanced and less stressful learning environment for students [32].

Policy Interventions

Government initiatives are crucial in raising awareness about student mental health and suicide prevention. Launching national campaigns can help in this regard. Additionally, allocating funds specifically for mental health programs in schools and coaching centers can ensure these initiatives are well-supported and sustainable, leading to better mental health outcomes for students [32].

Creating a Supportive Educational Environment

Mentorship programs should be established where senior students or alumni can guide current aspirants, providing emotional support and practical advice. Creating safe spaces within educational institutions where students can relax, socialize, and take breaks from their studies can also contribute to a more supportive and balanced educational environment [32].

Crisis Intervention

Setting up 24/7 helplines specifically for students can provide immediate access to mental health support in times of crisis. Developing crisis response teams within institutions, trained to handle emergency situations involving students in distress, can ensure that students receive the help they need when they need it most. These measures can play a crucial role in preventing suicides and supporting students' mental health [33].

## Conclusions

Our study provides valuable insights into the epidemiology and risk factors associated with suicides among students preparing for competitive exams, particularly IIT JEE and NEET, in India. The findings underscore the critical vulnerability of students in this high-pressure environment, with a significant proportion of suicides occurring among those in the 15-20 age group, particularly male students. To address this urgent issue, a multifaceted approach is needed, encompassing educational reforms, enhanced mental health support services, and community-based interventions to reduce academic stress and foster resilience among aspirants. Key strategies include integrating regular mental health screenings in educational institutions, providing accessible counseling services, implementing stress management workshops, and promoting a balanced, holistic approach to education that values creativity and critical thinking. Additionally, targeted interventions should be developed to address the unique challenges faced by male students and students from specific regions, considering socio-cultural and regional differences. This study also highlights the need for further research into socio-cultural factors, gender disparities, and regional differences that contribute to suicide risk among students, which will help refine strategies and interventions. These areas of future research will further guide the development of targeted and effective solutions to mitigate suicide risks. By framing these findings within a global and regional context, we emphasize the urgency of adopting similar measures worldwide to tackle the growing mental health crisis among students. Immediate action is essential to mitigate the suicide risks faced by students preparing for competitive exams and to safeguard their mental well-being for a brighter future.
